# Quantifying Bulk Electrode Strain and Material Displacement within Lithium Batteries via High‐Speed Operando Tomography and Digital Volume Correlation

**DOI:** 10.1002/advs.201500332

**Published:** 2015-12-18

**Authors:** Donal P. Finegan, Erika Tudisco, Mario Scheel, James B. Robinson, Oluwadamilola O. Taiwo, David S. Eastwood, Peter D. Lee, Marco Di Michiel, Brian Bay, Stephen A. Hall, Gareth Hinds, Dan J. L. Brett, Paul R. Shearing

**Affiliations:** ^1^ Electrochemical Innovation LabDepartment of Chemical EngineeringUniversity College LondonTorrington PlaceLondonWC1E 7JEUK; ^2^ Division of Solid MechanicsLund University221 00LundSweden; ^3^ ESRFThe European Synchrotron71 Rue des Martyrs38000GrenobleFrance; ^4^ Synchrotron Soleil, L'Orme des MerisiersSaint‐Aubin91192Gif‐sur‐YvetteFrance; ^5^ Manchester X‐ray Imaging FacilitySchool of MaterialsUniversity of ManchesterOxford RoadManchesterM13 9PLUK; ^6^ Research Complex at HarwellHarwell Oxford, DidcotOxfordshireOX11 0FAUK; ^7^ School of MechanicalIndustrial and Manufacturing EngineeringOregon State UniversityCorvallisOR97331‐6001USA; ^8^ National Physical LaboratoryHampton RoadTeddingtonMiddlesexTW11 0LWUK

**Keywords:** degradation, digital volume correlation, lithium batteries, operando imaging, X‐ray computed tomography

## Abstract

Tracking the dynamic morphology of active materials during operation of lithium batteries is essential for identifying causes of performance loss. Digital volume correlation (DVC) is applied to high‐speed operando synchrotron X‐ray computed tomography of a commercial Li/MnO_2_ primary battery during discharge. Real‐time electrode material displacement is captured in 3D allowing degradation mechanisms such as delamination of the electrode from the current collector and electrode crack formation to be identified. Continuum DVC of consecutive images during discharge is used to quantify local displacements and strains in 3D throughout discharge, facilitating tracking of the progression of swelling due to lithiation within the electrode material in a commercial, spiral‐wound battery during normal operation. Displacement of the rigid current collector and cell materials contribute to severe electrode detachment and crack formation during discharge, which is monitored by a separate DVC approach. Use of time‐lapse X‐ray computed tomography coupled with DVC is thus demonstrated as an effective diagnostic technique to identify causes of performance loss within commercial lithium batteries; this novel approach is expected to guide the development of more effective commercial cell designs.

## 1 Introduction

This is an open access article under the terms of the Creative Commons Attribution License, which permits use, distribution and reproduction in any medium, provided the original work is properly cited.

Due to their durability, high specific energy, and high energy density,^1^ lithium primary batteries are the most favored energy storage technology for demanding applications where recharging is impractical, e.g., emergency transmitters^2^ and safety, military and security devices. Due to their widespread application in mission‐critical systems, understanding the safety and reliability of primary battery operation is of significant importance, as highlighted by recent high‐profile failure events in the aerospace sector.^2^

Lithium batteries are becoming increasingly energy dense^3^ and new generation electrode materials^4^ are being developed to meet today's demanding applications. However, many functional electrode materials undergo a significant volume change during the insertion of Li^+^ ions^5^, ^6^ that can induce high local strains and severe mechanical degradation^7^ leading to capacity fade and reduced performance. Furthermore, understanding the interaction between electrochemically active materials and the mechanical design of commercial batteries is becoming increasingly important particularly with the introduction of advanced materials with high specific capacities and a large degree of motion and morphological evolution during lithiation.^8^, ^9^ This displacement of active materials can lead to contact loss between the current collector and electrode material which is thought to be one of the primary mechanisms for increased cell impedance during cycling.^10^, ^11^ Numerous studies have been performed to tackle the issue of high strain and mechanical degradation on both the micro and macro scale, including the development of strain models to determine the particle fracture conditions^9^, ^12^, ^13^ and the reduction of contact losses by increased applied pressure.^14^ However, little data are available to help understand the dynamic mechanisms associated with morphological evolution of active materials and their interaction with the mechanical design of commercial cells.

Various in situ characterization techniques have been used to track lithium insertion and morphological changes of electrode materials during operation.^15^ Techniques such as soft X‐ray absorption spectroscopy^16^ and neutron imaging^17^ have previously been used to track the lithiation of electrode materials during operation, but both are restricted to 2D measurements for fast dynamic processes due to their low temporal resolution and consequent high acquisition times for 3D images. In situ X‐ray diffraction (XRD) has also been used to monitor the progress of lithiation/delithiation of active materials inside operating lithium–ion batteries by studying their structural changes.^18^ Additionally, coherent XRD imaging has been shown to be a powerful tool for characterization of strain evolution in the crystal lattices of individual electrode particles on the nanoscale.^19^ However, whilst XRD measurements are useful for tracking reaction mechanisms, they provide no information on the impact of the resulting structural changes on the morphology of the bulk material.

X‐ray computed tomography (CT) is used as a non‐invasive diagnostic tool to quantitatively analyze the morphology of materials in 3D. Synchrotron radiation sources can provide the high photon flux and brilliance necessary to achieve high resolution 3D images over short periods of time^20^ presenting the possibility for tracking rapid structural evolution processes of active materials in 3D and in real time.^21^ The evolution of 3D microstructures of electrode materials has mostly been quantified previously via X‐ray CT by comparing before and after morphological evolution.^22^, ^23^ However, time lapse tomography is increasingly used to track the evolution of materials and the coupling of such imaging with image correlation techniques, such as 3D digital volume correlation (DVC),^24^ provides a powerful diagnostic tool in materials research.^23^, ^25^, ^26^, ^27^

In this study, we capture the evolution and quantify the strain in the bulk architecture of the MnO_2_ electrode material in a commercial primary Li/MnO_2_ lithium battery during a high rate, constant resistance discharge. As Li/MnO_2_ cells have a high discharge rate capability (they are often used for high drain applications), we use the high speed imaging capability of beamline ID15A^28^ at ESRF—The European Synchrotron. Furthermore, we use two different DVC software packages developed by Hall et al.^25^, ^26^ and Bay et al.^24^ to track the transient local displacements of the MnO_2_ electrode in 3D as lithiation‐induced dilation occurs. 3D displacement fields generated using continuum DVC are used to derive 3D strain profiles of the electrode material throughout operation. Additionally, the electromechanical effect of the dilating electrode on the structural integrity of the cell architecture is assessed. The extent of unraveling of the current collector is quantified using a separate DVC approach and related to the temporal continuum strain field evolution. The importance to commercial cell designs of accommodating the changes in cell architecture during operation is highlighted, and this new approach of combining high speed tomography and DVC is demonstrated as an effective technique for identifying causes of performance loss within commercial batteries.

## 2 Materials and Methods

### 2.1 Operando X‐Ray Tomography

This study focuses on a commercial, spiral wound, Duracell CR2 battery^29^ which was discharged between 3 and 2 V during high speed X‐ray CT imaging. Experiments were performed at beamline ID15A at ESRF – The European Synchrotron, known for its high speed and high resolution imaging capability.^21^, ^28^ The high photon flux and brilliance of beamline ID15A generates the spatial and temporal resolution required to identify the fine granular structure of the electrode material within the short time scales over which significant material evolution occurs during battery operation. Furthermore, the DVC displacement and strain analyses subsequently performed rely on correlating consistent, identifiable grain texture in consecutive images; therefore, DVC analysis requires images to be acquired at sufficiently short intervals to avoid excessive changes in the images.

**Figure 1:**
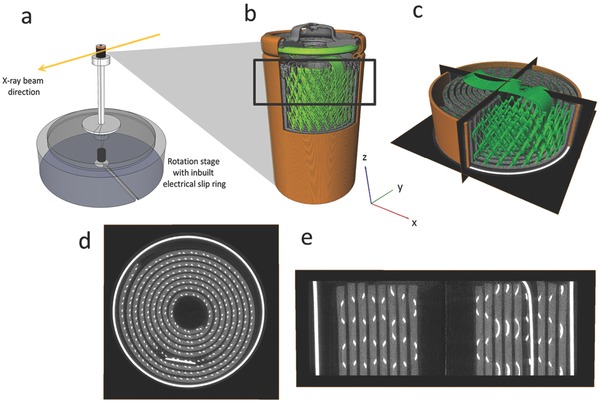
a) Experimental setup showing rotation stage and in‐built electrical slip ring connection. b) Stitched reconstruction of a full commercial Duracell CR2 battery showing the casing (orange), current collector mesh connected via a tab to the terminal (green), and MnO_2_ electrode (gray). The black square represents the region which was scanned during continuous X‐ray CT. c) Reconstructed tomogram of the section captured during continuous X‐ray CT with orthoslices in the *XY*, *YZ* and *XZ* planes. d) Isolated *XY* slice showing battery casing and current collecting mesh (white) and MnO_2_ electrode (gray). e) Isolated *XZ* slice.

The Duracell CR2 battery was imaged in a 76 keV monochromatic X‐ray beam with a field of view (FOV) of 8.6 mm × 8.7 mm and a pixel size of 10.87 μm (794 × 800 pixels), corresponding to half of the cell (see **Figure** **1**). The sample stage rotation axis was placed at the edge of the FOV, such that by a 360° rotation it was possible to image the entire sample.^30^ Each tomogram was composed of 2 × 2000 half projections. Tomograms were captured with angular increments of 0.09° between images. A high speed camera, PCO Dimax (PCO AG, Germany), with an exposure time of 0.7 ms was used during imaging. ­Consequently, the acquisition time of each tomogram was 2.8 s which was sufficiently fast to avoid image artifacts caused by sample motion during acquisition. During continuous X‐ray CT, tomograms were captured every 40 s which included image acquisition time and data transfer to the ESRF central servers. No heating of the cell due to incident X‐ray radiation was observed.

The CR2 battery was discharged at a constant resistance of 2.75 Ω during continuous X‐ray CT imaging. The electrical connection between the battery and constant resistance circuit was maintained through use of an electrical slip ring (P4+ Compact Slip Ring, Moog, UK) built into the ID15A rotation stage (ABR1000, Aerotech, USA) (Figure 1a), which allowed continuous rotation while maintaining an electrical connection. Copper foil was pressed against the terminals of the battery using two copper tabs tightly wrapped with electrical tape. An electrical cable was soldered to each of the copper tabs. For the electrical cable connected to the positive terminal (top in Figure 1b) a relatively X‐ray transparent copper mesh was used to minimize beam hardening artifacts as the cable passed in front of the field of view during rotation. The radiographs were reconstructed into 8.7 mm high cylindrical sections (tomograms) of the cell (Figure 1b,c) using standard reconstruction methods.^31^

The reconstructed tomograms were processed using Avizo Fire 7 (FEI VSG, France) whereby the 3D images could be separated into 2D planar slices, as shown in Figure 1c–e. Highly attenuating materials appear white in the tomograms, while less attenuating materials are darker in appearance. Material phases of interest were isolated based on grayscale values using Avizo's segmentation editor. The casing and steel current collecting mesh displayed in Figure 1d,e are highly attenuating and are displayed as white, and the Li*_x_*MnO_2_, which consists of a slurry coated onto the spiral wound current collecting mesh, is displayed as light gray. Lithium metal and separator material are poorly attenuating at 76 keV and difficult to distinguish, but are situated between the spiral wound MnO_2_ anode layer. The copper mesh attached to the outside of the battery casing was cropped out of Figure 1d. The 3D image files were stored in 32‐bit .raw file format for volume correlation.

### 2.2 Continuum DVC and Strain Field Evolution

**Figure 2:**
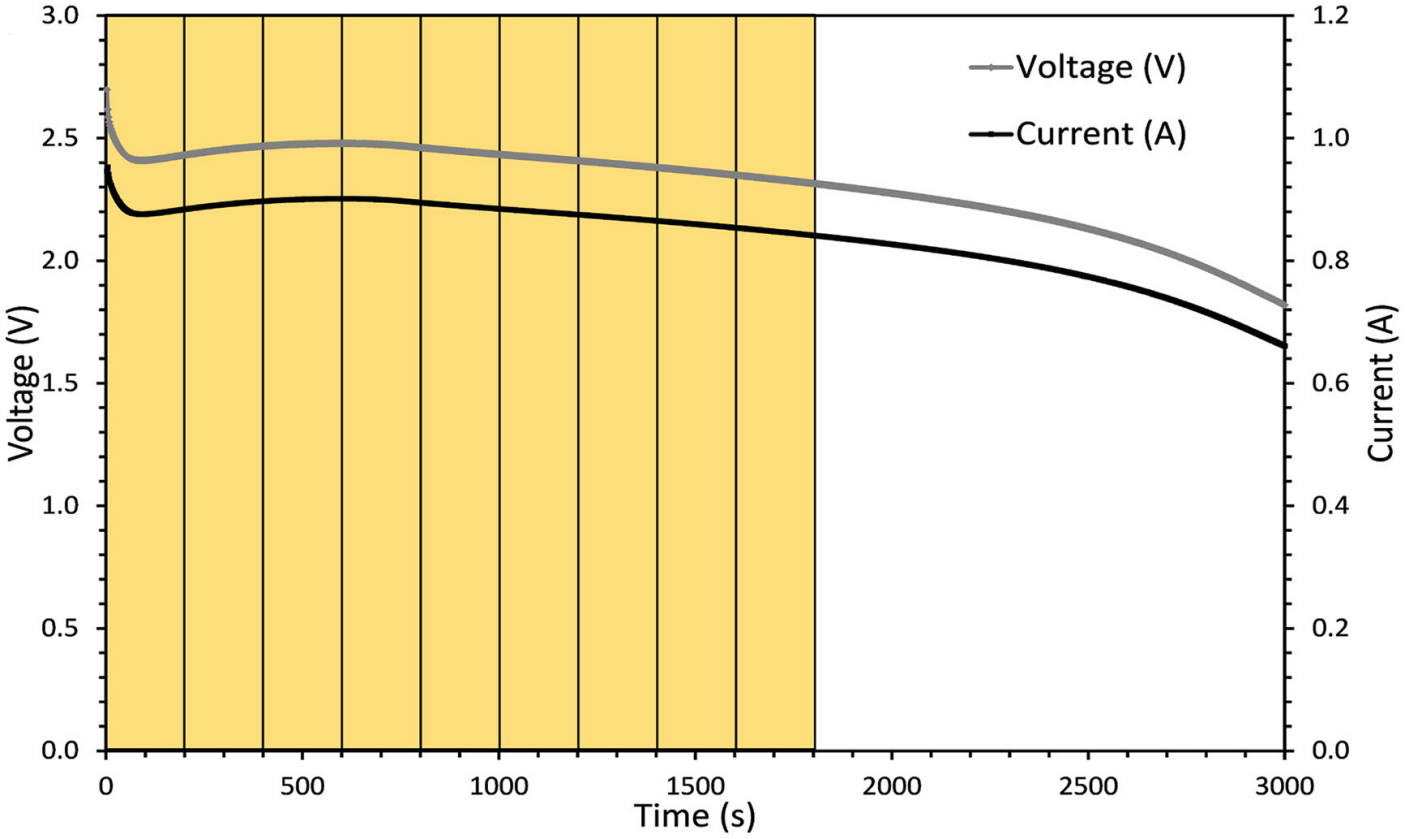
Discharge curve of the CR2 cell at constant resistance of 2.75 Ω. The yellow segments indicate the time between consecutive volume images which were correlated using DVC.

In this study, the CR2 cell is discharged from its fully charged state (3 V) to below 2 V, as shown in **Figure** **2**. The MnO_2_ is reduced from tetravalent to trivalent state to form Li*_x_*MnO_2_ (where 0 ≤ *x* ≤ 1) as the Li^+^ ion inserts into the MnO_2_ lattice.^32^, ^33^ 3D displacement measurements of textured materials can be performed by comparing X‐ray CT images via DVC.^24^, ^26^, ^34^ The continuum DVC in this work uses TomoWarp2 code developed between the Universities of Lund and Grenoble building on the previously developed code, TomoWarp of Hall.^25^, ^26^, ^35^ In the current context, continuum DVC refers to the displacement calculation over a 3D grid of node points that are then used to determine 3D tensor strain fields following continuum mechanics.

The DVC approach can be described briefly as having the following main steps:

- A regular 3D grid of nodes was defined over the CT image volume, and a “correlation window” was centered on each node for the correlation analysis. In the current study, the grid spacing of the nodes was 5 voxels in each direction and the correlation window had a volume of 15 × 15 × 15 voxels.
- The displacement for each node was derived by identifying the 3D translation that maximizes the cross‐correlation of the gray‐level data within the correlation window in the nondeformed image and the translated correlation window in the deformed image. The displacement field so determined had a precision of 1 voxel in each direction.
- Subvoxel resolved translation vectors were determined based on the maximum of the interpolated correlation coefficient for a range of displacements around the initial integer voxel value.
- Calculation of the strain tensor was based on the gradient of the displacements over eight‐point cubic elements of neighboring nodes.

Following the above continuum DVC approach, the evolution of the Li*_x_*MnO_2_ electrode architecture was quantified in 3D by correlating successive tomograms. Tomograms were captured every 40 s during discharge and increments between sequential tomograms which showed significant material displacement were chosen for correlation. The discharge profile of the CR2 battery in Figure 2 shows image increments of 5 between the reference and deformed images for DVC, which corresponds to a difference of 200 s during discharge. Note that the images were cropped to 1487 × 1387 × 501 voxel volumes to remove artifacts at the edges of the FOV and to isolate the regions of interest. Also, the size of the search windows within which the 3D translation was searched in the DVC varied between each increment and, due to its high impact on computation time, was optimized before the entire volumes were loaded.

For each volume correlation the correlation coefficient of the Li*_x_*MnO_2_ material was >0.97 (Figures S1–S9, Supporting Information); whereas poor correlation was observed for all other materials. DVC analysis points that gave a normalized cross correlation coefficient of less than 0.97 were filtered out.

### 2.3 Current Collector Displacement Measurement

As a separate measurement, the DVC methodology was used to determine the deformation of the collector mesh during battery discharge. The current collector is a metallic mesh of diamond pattern, embedded within the electrode material, and spirally wound within the battery case. The mesh contains distinct nodes where individual wires meet, creating unique features that can be tracked as deformation occurs. Three layers of 176 nodes were identified within the battery, and their centers located approximately by standard imaging methods. Each point formed the center of a rectangular 40 × 40 × 80 voxel subvolume, large enough to contain the complete connection, but small enough to prevent interaction between layers of the scroll. Subvolumes were extracted from imaging of a charged battery and tracked within imaging of the discharged state. Subvoxel translational and rotational degrees of freedom were activated for a zero normalized sum of square difference objective function with tricubic spline interpolation and a typical coarse‐fine search strategy was employed. Convergence tolerance was tightened until all points tracked successfully and displacement values stabilized. Deformations were visualized with 3D vector plots showing motion of points within the three layers of junction points.

## 3 Results and Discussion

### 3.1 Evolving Morphology of Li*_x_*MnO_2_ during Lithiation

The volume expansion observed during lithiation of Li*_x_*MnO_2_ can cause severe mechanical degradation on both the micro and macro scale. The exact volume expansion of the Li*_x_*MnO_2_ is difficult to determine via threshold segmentation due to the change in attenuation of Li*_x_*MnO_2_ as it lithiates and the overlapping grayscale of different materials within the cell. Additionally, individual crystallites tend to break up during discharge^32^ and any increased electrode material porosity would not be detected at the voxel resolution used here, which is not fine enough to identify individual Li*_x_*MnO_2_ particles. However, an estimate of 20% volume expansion due to lithiation was determined by comparing the quantity of the grayscale threshold associated with the electrode material before and after discharge. Over a single discharge at low operating voltages (2–3 V) it is not expected that dissolution of Li*_x_*MnO_2_ has any significant effect on the observations in this work.

**Figure 3:**
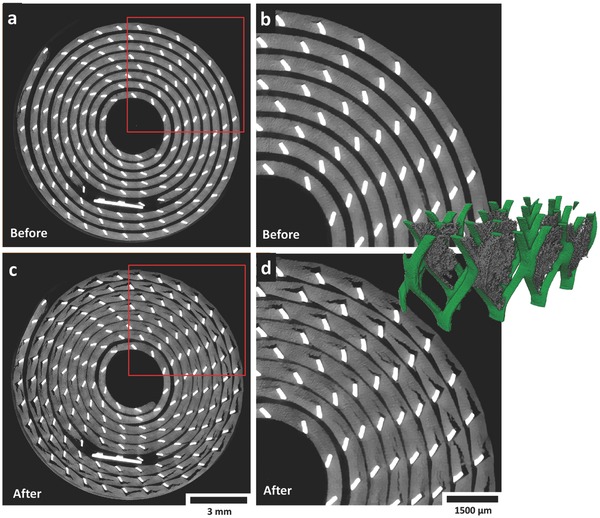
a) Gray scale *XY* slice (horizontal cross section) from a tomogram captured before discharge; the red square indicates the region of interest with which the neighboring image is associated. b) Enlarged view of the *XY* slice showing pristine electrode before discharge. c) Gray scale *XY* slice from a tomogram captured after discharge; the red square indicates the region of interest with which the neighboring image is associated. d) Enlarged view of the *XY* slice showing electrode after discharge. Inset: Section of the current collecting mesh showing a 3D view of the crack openings. Movies S1 and S2 (Supporting Information) show the evolution of the slices in the *XY* and *ZX* plane in real time.

**Figure** **3** shows horizontal slices taken from tomograms of a CR2 cell before and after discharge. Two movies showing the intermediate stages in real‐time are presented as time‐stamped horizontal (Movie S1, Supporting Information) and vertical (Movie S2, Supporting Information) slices of the CR2 cells during discharge. In Figure 3 and Movies S1 and S2 (Supporting Information), the Li*_x_*MnO_2_ spiral wound layer expands and the thickness of the lithium region is seen to diminish during the lithiation process. The battery casing is included in these movies to observe any interaction between the evolving active materials and the casing.

Detachment of the Li*_x_*MnO_2_ from the current collecting mesh, as evidenced by the cracks emanating in the anticlockwise direction in Figure 3, occurs as a consequence of the spiral wound materials not being tightly wound or compressed. Additionally, Movie S1 (Supporting Information) reveals that the evolving architecture of the Li*_x_*MnO_2_ electrode causes a slight degree of unraveling of the spiral wound materials, a consequence of the mechanical energy associated with the electrode‐active material.^6^

The internal unraveling of the electrode layers further contributes to crack propagation via the strain induced from the movement of the current collecting mesh against the active material, explaining the increased severity of cracking with distance from the center of the cell observed in Figure 3. This detachment of the Li*_x_*MnO_2_ from the current collecting mesh results in a significant reduction in interfacial area between the current collecting mesh and the active material. The inset in Figure 3 shows a 3D reconstruction of a small section of the mesh with isolated crack regions; the significant decrease in interfacial area between the electrode material and the current collecting mesh is clearly visible.

Delamination of electrode materials from the current collector is a well‐known cause of increased cell impedance and capacity fade in commercial cells.^36^ The interfacial area available for charge transfer is reduced leading to an increase in ohmic resistance and consequently local heating. However, given that the CR2 cell is a primary cell and capacity fade with cycling is not a cause of concern, the unconstrained expansion of the Li*_x_*MnO_2_ and the formation of cracks within the Li*_x_*MnO_2_ material may also benefit the performance of the cell by exposing additional Li*_x_*MnO_2_ surface area to Li‐ion transfer. The experiment was repeated for a slower discharge with a constant resistance of 4.5 Ω; the time‐stamped slices show similar behavior but over a longer period of time. The discharge plot and evolution movies are provided in Figure S10 and Movies S3 and S4, Supporting Information.

DVC was applied to consecutive images to determine the 3D strain profiles caused by lithiation of the electrode and to explore the electrochemically induced strain that causes unraveling of the current collecting mesh.

### 3.2 Quantification of Temporal Local Strain

**Figure 4:**
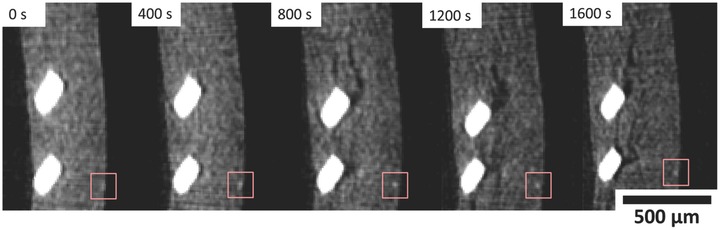
Magnified sections of an *XY* slice from consecutive tomograms. The red squares indicate the size of the correlation windows (but in 3D cubes) for feature tracking and contain a consistent grain feature (white spot), typical of one which would be tracked for displacement measurements during DVC.

Continuum displacement DVC between consecutive tomographs was applied to plot the evolution of 3D displacement and strain profiles. Consistent grain detail is required between consecutive images to measure continuum displacement with a high correlation coefficient. An example of the fine detail present in the tomography images is shown in **Figure** **4** where the texture is provided by the electrode microstructure (particle size distribution of Li*_x_*MnO_2_, binder and conductive material, and electrolyte‐filled pore phase). The correlation window was set as a cube of 15 voxels which, with a voxel resolution of 10.87 μm, tracks grain detail up to 163 μm^3^. The distance between nodes was set as 5 voxels or 54.35 μm.

**Figure 5:**
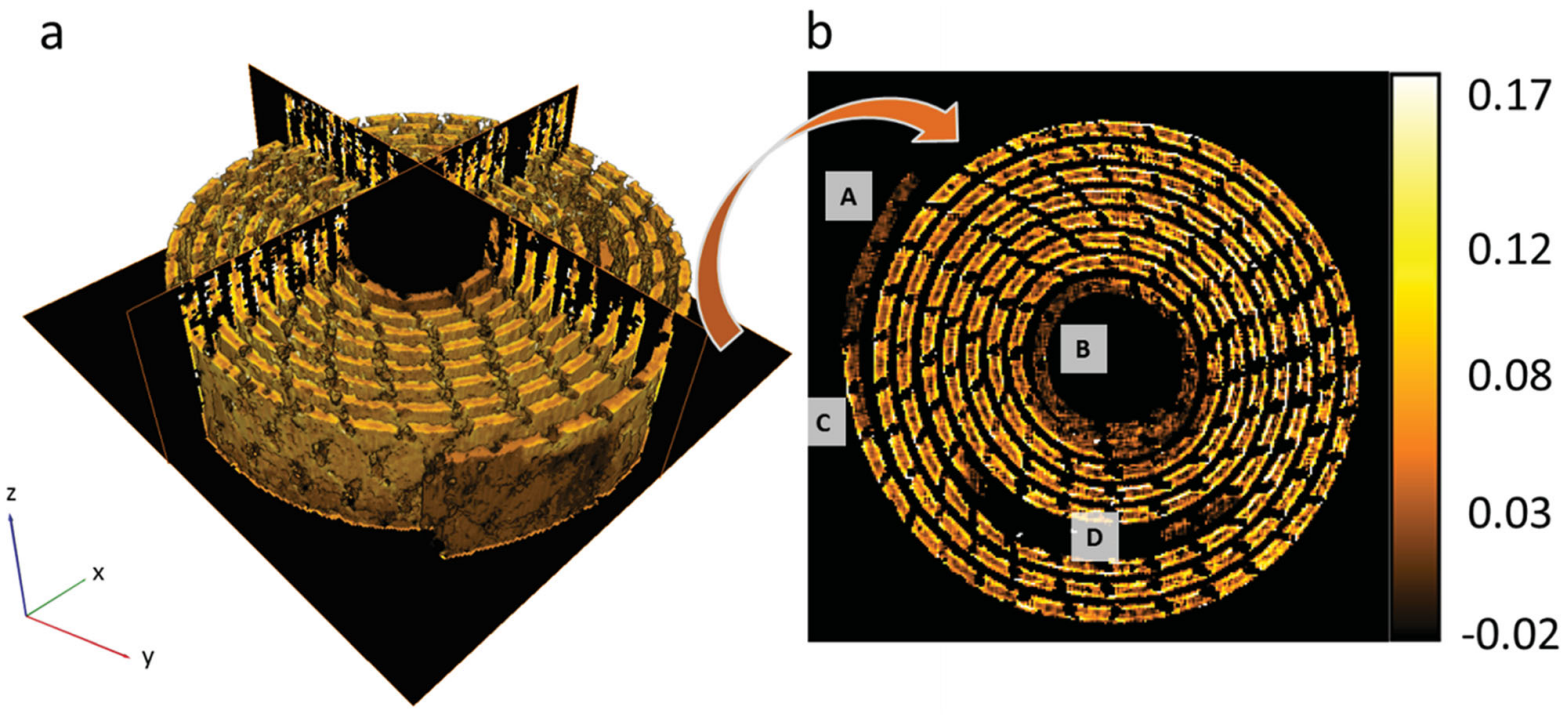
a) 3D reconstruction of volumetric strain extracted from DVC of images 200 and 400 s into discharge, including ortho‐slices in the *XY*, *YZ* and *XZ* planes. b) Extracted *XY* slice showing volumetric strain profile. Labels A and B identify inactive regions at the outer and inner ends of the Li*_x_*MnO_2_ layer, label C highlights a region in which lithiation occurs from one side only and label D shows a dark region from which the current collecting tab was filtered.

During lithiation, it is expected that the induced volume expansion of Li*_x_*MnO_2_ will be observed as bulk deformation of the active electrode layer on the submillimeter scale via interaction and displacement of the Li*_x_*MnO_2_ active crystallites, binder and the conductive carbon substrate. The continuum DVC provided averaged displacement profiles between 3D images which were 200 s apart during discharge. Displacements were plotted in 3D and the associated strain maps were generated by differentiating the vector displacements with respect to the *X*, *Y*, and *Z* directions. **Figure** **5**a shows a 3D reconstruction of a color map of volumetric strain of the Li*_x_*MnO_2_ electrode measured between 200 and 400 s into discharge. Correlation windows (individual voxels in Figure 5) which showed a poor correlation (≤0.97) were filtered out of the displacement profiles by replacing the voxels with not‐a‐number (NaN) value, creating a sparse matrix.

Clear strain features are observed which correspond to the local extent of dilation induced by lithiation over the correlated time period; for example, the ends of the spiral wound Li*_x_*MnO_2_ electrode labeled as regions A and B in Figure 5b exhibit no activity. Upon deconstruction of the cell it was found that the intermediate lithium metal foil stops before reaching the end of the Li*_x_*MnO_2_ layer which would explain the inactive regions (areas of low strain in Figure 5b, labeled A and B) at the inner and outer ends of the Li*_x_*MnO_2_ layer. At region C in Figure 5b, lithiation is seen to occur on one side of the Li*_x_*MnO_2_ layer only. The one‐sided activity around this region indicates that the lithium metal foil extends further on the outside of the electrode layer than on the inside. The area surrounding region D in Figure 5b is where the current collecting tab shown in Figure 1b,c travels down through the cell. The current collecting mesh and tab were filtered out of this image and are tracked separately; hence there is a large black region where the current collecting tab would otherwise be.

**Figure 6:**
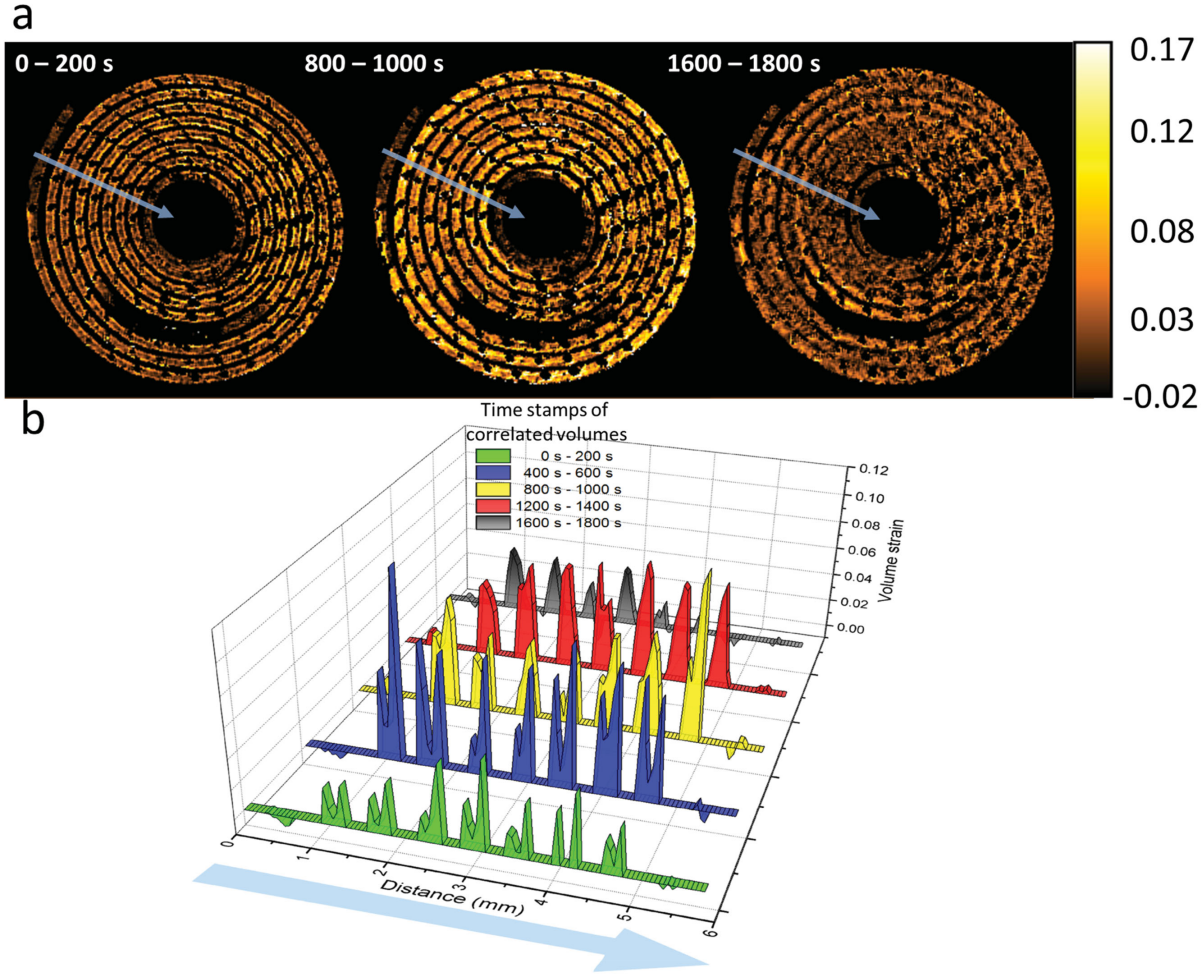
a) *XY* slices from the 3D volume strain images corresponding to DVC of the tomographs taken at the labeled times. Movie S5 (Supporting Information) shows the evolution of the strain profiles for consecutive correlated volumes 200 s apart during discharge. The average strain is measured across the five voxel wide blue lines from the outside to inside of the electrode layer (direction of arrows). b) Time series plot showing strain values which are averaged across five voxel wide lengths, shown as blue lines in the *XY* slices from the 3D strain profiles. Distance is plotted from outside to inside of the battery spiral.

Movie S5 (Supporting Information) shows the evolution of volumetric strain of the *XY* slice shown in Figure 5b where each frame displays the strain of consecutive correlated volumes 200 s apart during discharge. **Figure** **6**a shows selected *XY* slices from the volumetric strain profiles at initial, mid, and end stages of discharge, and Figure 6b shows a plot of the time dependent strain profiles across several of the electrode layers from beginning to end of the discharge, averaged across a 5 voxel wide line (blue lines in Figure 6a). The low correlation voxels which were previously given NaN values were reassigned a value of zero when plotting the graphs in Figure 6b. Initially a region of high strain is observed around the surface and subsurface of the active Li*_x_*MnO_2_ layer, indicating a mass transport limited electrode and consequently, a concentration gradient of lithium across the active layer. The nonuniform dilation caused by the lithium concentration gradient across the thick electrode challenges the mechanical integrity and can result in crack evolution and particle isolation. During the early stages of discharge, very little strain is observed around the inner regions of the Li*_x_*MnO_2_ layers.

As lithiation progresses deeper into the active material, the dilation front progresses toward the center of the electrode layer, and hence the strain in this region begins to increase, which is already evident between 400 and 600 s in Figure 6b. As time progresses, the concentration inside the layers becomes larger but the overall strain and strain gradient reduces as the rate of lithiation‐induced expansion and displacement of the Li*_x_*MnO_2_ decreases. This results in nonuniform insertion stresses on the microscale.^13^ However, due to the flexibility of the electrode material on the submillimeter scale, where the displacement of microparticles can change the porosity of the bulk material,^27^ the strain observed on the bulk material is not expected to be representative of the local strain on the active Li*_x_*MnO_2_ crystallites.^13^

A significant difference in strain is evident between both sides of each electrode layer, as identified in the twin peaks at these locations in Figure 6b. During operation lithium metal is consumed from both sides of the electrode and diffuses into both sides of the Li*_x_*MnO_2_; however, the strain observed on the inner side of the electrode layer (right of the twin peaks in Figure 6b) is generally higher than that on the outer side (left of the twin peaks in Figure 6b). This is most likely an attribute of the positioning of the current collecting mesh in the electrode material.^37^ As seen in Figure 3 and more closely in Figure 4, the current collecting mesh is positioned close to the inner edge of the Li*_x_*MnO_2_ layer, an artifact of the manufacturing process in which the mesh is placed on a surface and coated with active material. This results in the effective electrode thickness (and therefore resistance) being different on each side of the current collecting mesh.^38^ The effective electrode thickness is greater when the tortuous path of the lithium ions through the porous electrode material is considered. The differing effective electrode thicknesses arising from the placement of the current collector prevent optimized utilization of electrode material. A large gradient in lithium concentration can cause a drop in the electrode potential and can result in underutilized electrode material by the time the cell reaches the cut‐off voltage,^37^, ^39^ resulting in reduced cell capacity. Hence, thick electrodes can result in a significant potential drop between the surface of the electrode layer and the current collector during operation, which highlights the importance of optimizing electrode thickness for particular operating conditions.

In battery modeling the current density is given by the reaction current density at the surface of the electrode particles, which is determined by the surface lithium diffusion flux. The surface area of the outer side of the electrode layer is greater than the surface area of the inner side of the electrode due to its larger radius of curvature. If the current density is assumed to be uniform on both sides of the current collecting mesh, then with the increased surface area on the outer side of the Li*_x_*MnO_2_ material it is expected that the local current density and hence activity would be lower, which is consistent with a lower strain on the outside of the Li*_x_*MnO_2_ layers in Figure 6b.

**Figure 7:**
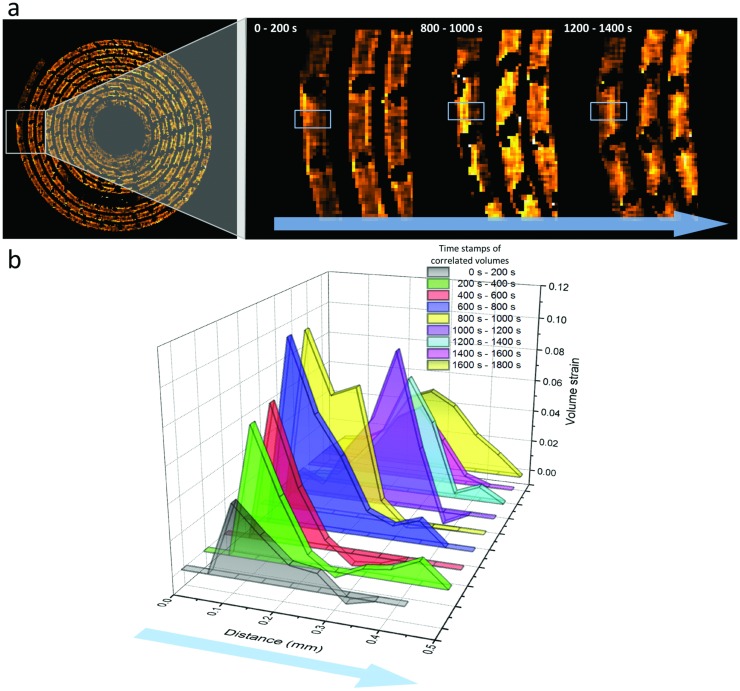
a) Enlarged views of a region of interest at different states of charge, where lithiation occurs on one side only (outside to inside). The progression of lithium into the Li*_x_*MnO_2_ is observed by the strain front propagating through the material during discharge, as seen in the regions highlighted by the gray boxes. The evolution of this process can be seen in Movie S5 (Supporting Information). b) Averaged strain in the vertical direction of the boxed regions of interest showing the evolution of strain across the Li*_x_*MnO_2_ layer during one‐sided lithiation. The blue arrows indicate the direction of measurement.

On the outer end of the Li*_x_*MnO_2_ layer the one‐sided activity in **Figure** **7**a indicates that the lithium foil ends at different points between the outside and inside of the Li*_x_*MnO_2_. At this location, lithium diffuses into the Li*_x_*MnO_2_ in one direction only, radially inward. The progression of lithiation can be seen from the strain front traveling through the electrode material during operation, as shown in Figure 7a. Figure 7b shows a plot of the strain across a labeled region of interest calculated from consecutive correlated volumes during discharge. Between 0 and 600 s during discharge, the strain profile shows an increasing strain around the subsurface region of the electrode as lithium inserts into the Li*_x_*MnO_2_ from the outer surface. As the lithiation progresses, the strain front travels deeper into the active material. The observed strain decreases near the end of discharge as the Li*_x_*MnO_2_ becomes increasingly concentrated with lithium and the rate of lithiation (and particle dilation) slows down. The traveling strain front is indicative of the progressing lithium concentration distribution within the electrode during discharge, which emphasizes the potential for DVC as a model validation technique for evolving lithium concentration in active materials during operation.^39^

### 3.3 Mechanical Influence of Active Materials

**Figure 8:**
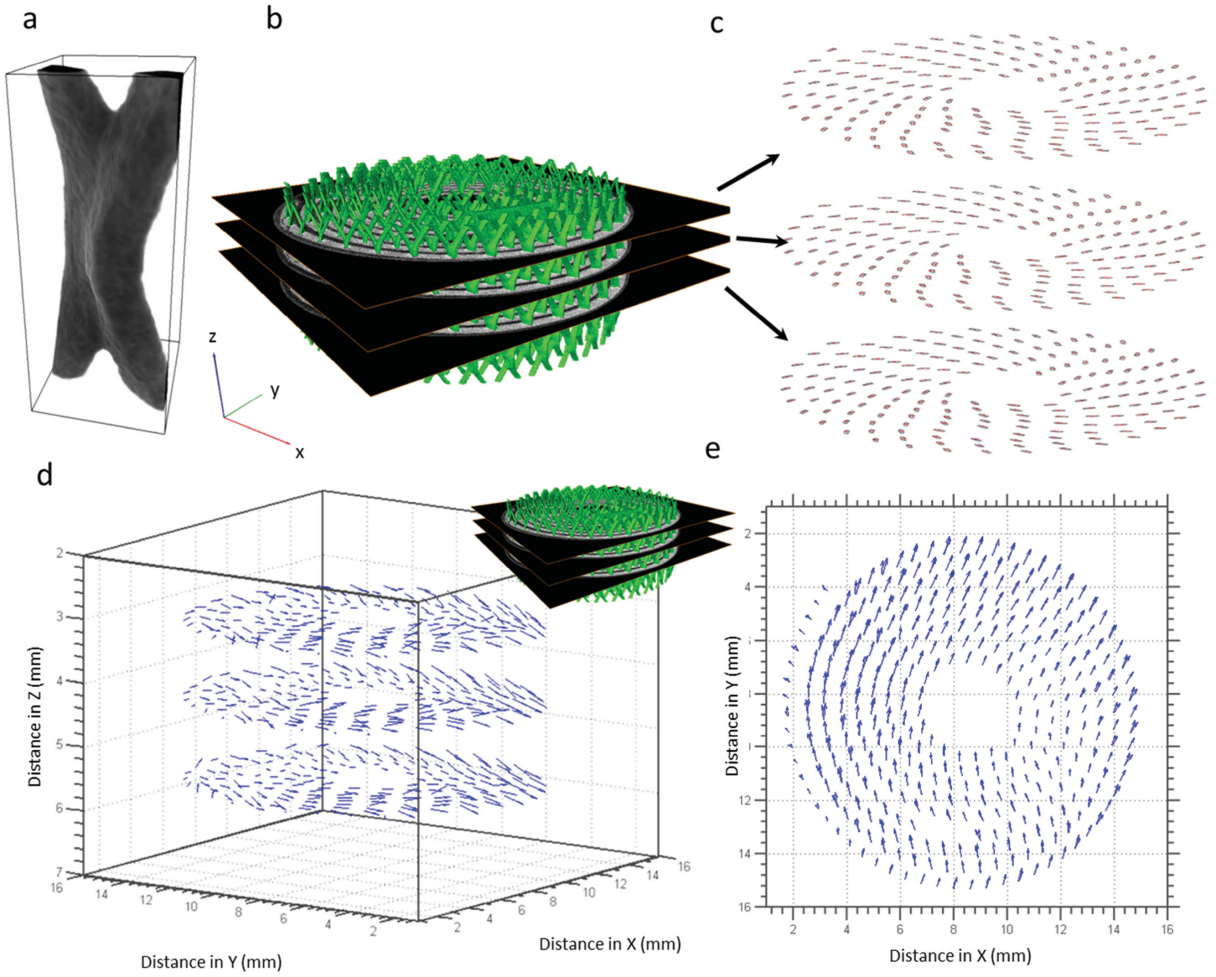
a) A typical collector grid junction point and the rectangular subvolume used for DVC. The subvolume is 40 × 40 × 80 voxels in size. b) 3D reconstruction of the current collecting mesh with *XY* slices for point tracking. c) Binarised current collector points selected for point tracking in three *XY* slices along the *Z* direction. d) Vector plot showing the magnitude and direction of translation of the current collector points in 3D. Most displacement occurs in the *XY* plane. e) Top‐down view of the vector plots in the *XY* planes showing displacement of the rigid current collector points in the *X* and *Y* directions.

The measurements above have demonstrated that the evolving Li*_x_*MnO_2_ architecture displaces the rigid current collecting mesh which detaches from the electrode material. This leads to increased contact resistance between the electrode and current collector which can significantly affect the performance of the cell^14^ and cause capacity loss due to electrical isolation.^40^ In Movie S1 (Supporting Information), the Li*_x_*MnO_2_ electrode is seen to expand against the shell of the casing on one side only (bottom of Movie S1, Supporting Information); consequently, the spiral wound layers shift toward the vacant side of the cell. The shift of the current collecting mesh was quantified by using a point tracking method developed by Bay et al.^24^ whereby subvolumes containing consistent textures are tracked between subsequent images through correlation methods. Subvolumes of the current collecting mesh of 40 × 40 × 80 voxels in size (**Figure** **8**a) were extracted from 176 manually selected points on three layers along the current collecting mesh (Figure 8b,c). These subvolumes were tracked between images associated with the charged and discharged state of the battery. The displacement of the current collecting mesh resulting from the electrode expansion is quantified and the displacement vector plots are shown in Figure 8d. Most of the displacement occurs in the *XY* plane, whereas very little displacement is observed in the vertical *Z* direction. Figure 8e shows the point displacements in the *XY* plane where displacements up to 0.8 mm are observed. As seen in Movie S1 (Supporting Information), the spiral wound layers are only in contact with one side of the casing; as the electrode expands during lithiation the contents of the cell are pushed in the opposite direction, as shown in Figure 8e. The breakage of the Li*_x_*MnO_2_ electrode material resulting from displacement of the current collector can cause electrically isolated electrode sections as a result of loss of direct electrical contact with the current collector.^10^ Sufficient compression is necessary to hold the electrode together and help mitigate loss of contact with the current collector. However, as observed in this cell, the electrode layers are mostly unconfined allowing the active material to freely expand and unravel, which is detrimental to the structural integrity of the active layers.

## 4 Conclusions

DVC software has been applied to high speed operando X‐ray CT of commercial primary lithium batteries during discharge. By quantifying the movement of discrete sections of the electrode material between sequential images during discharge, a spatial and temporal map of material activity and strain profiles was generated. The progression of the lithiation front has been tracked by measuring the local displacement of electrode material during dilation for the first time which has provided valuable insights into the transient structural mechanics that take place within commercial cells during operation. For example, relatively inactive regions were identified and large concentration gradients within the electrode layers could be deduced from the evolving strain patterns, giving the capability to pinpoint inefficiencies and identify means through which the performance of the cell could be improved.

Although this cell is a primary cell, this novel diagnostic approach has provided a means of identifying numerous sources of performance loss, which may also be applied to rechargeable cells. The placement of the current collecting mesh to one side of a thick electrode during manufacturing has resulted in a significant variation in Li^+^ transport and dilation between sides of the electrode layer causing an uneven distribution of activity and strain. The cell was identified as being limited by Li^+^ transport since throughout the discharge the material dilation progressed from the surface of the electrode toward the current collector. This technique could also provide empirical evidence for whether optimum electrode thickness is achieved for particular operating conditions and assess the tension and compression effects associated with thick spiral wound electrodes.

This DVC approach also provides valuable information for multiphysics modeling of operating lithium batteries by providing real‐time, structural, and strain information in 3D. As demonstrated here, the interaction between evolving materials and their environment is imperative to accurately predict the performance of a particular cell design during operation, as crack formation and delamination can have a significant impact on the rate of aging and degradation of cells. Internal mechanics of a cell can also be elucidated and used to determine the tendency of a cell to incur capacity loss via breakage and isolation of electrode material. The extent of expansion, cracking, and delamination observed during discharge of the cell affects the local ionic diffusivity and electrical conductivity of the material.

To date, very few experimental studies have quantitatively captured the temporal local activity of electrode material within lithium batteries; in this work, the combination of the high‐resolution, high‐speed imaging capabilities of synchrotrons, and image correlation techniques, has been demonstrated as a powerful tool for identifying local strain, material displacement, and degradation within an operating cell. This approach could also be applied on the microscale to extract 4D strain profiles of individual electrode particles and be used as a tool for strain model validation. Information about local lithiation could be extracted from the correlation volumes by observing the material displacement fronts. The elucidation of the evolving active materials and their interaction with commercial mechanical designs is expected to guide the optimization of material use and commercial cell designs.

## Supporting information

As a service to our authors and readers, this journal provides supporting information supplied by the authors. Such materials are peer reviewed and may be re‐organized for online delivery, but are not copy‐edited or typeset. Technical support issues arising from supporting information (other than missing files) should be addressed to the authors.

Supplementary

Supplementary

Supplementary

Supplementary

Supplementary

Supplementary
