# Supplementary material for: Quantifying Bulk Electrode Strain and Material Displacement within Lithium Batteries via High‐Speed Operando Tomography and Digital Volume Correlation
Source: Adv Sci (Weinh). 2015 Dec 18;3(3):1500332. doi: 10.1002/advs.201500332 (PMC4991305; doi:10.1002/advs.201500332)
Supplement: Supplementary file 1 — Supplementary [file ADVS-3-1500332-s001.pdf]

Copyright WILEY-VCH Verlag GmbH & Co. KGaA, 69469 Weinheim, Germany, 2015.

Supporting Information

for *Adv. Sci.,* DOI: 10.1002/advs.201500332

Quantifying Bulk Electrode Strain and Material Displacement within Lithium Batteries via High-Speed Operando Tomography and Digital Volume Correlation

*Donal P. Finegan, Erika Tudisco, Mario Scheel, James B. Robinson, Oluwadamilola O. Taiwo, David S. Eastwood, Peter D. Lee, Marco Di Michiel, Brian Bay, Stephen A. Hall, Gareth Hinds, Dan J. L. Brett, and Paul R. Shearing**

Supplementary Figures

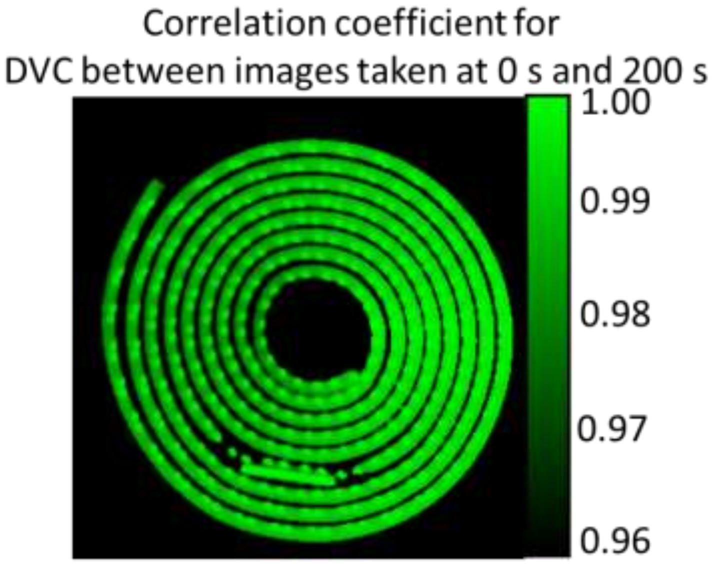

Supplementary Figure 1. *XY* slice from the correlation coefficient profile for DVC between images taken at 0 s and 200 s.

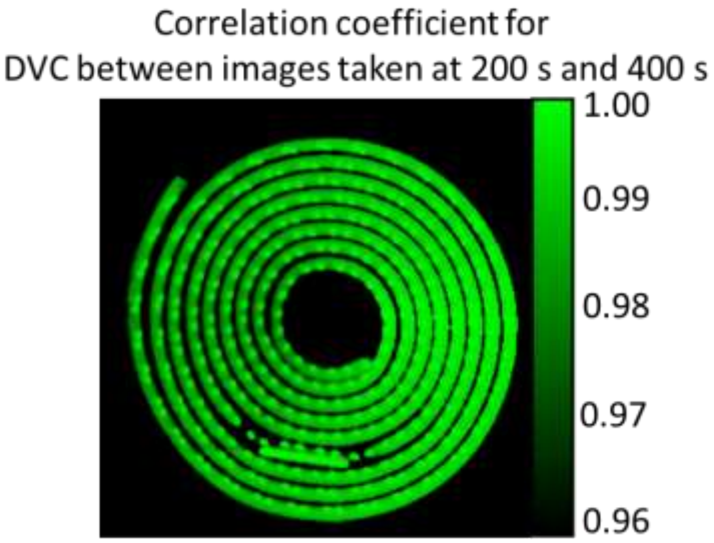

Supplementary Figure 2. *XY* slice from the correlation coefficient profile for DVC between images taken at 200 s and 400 s.

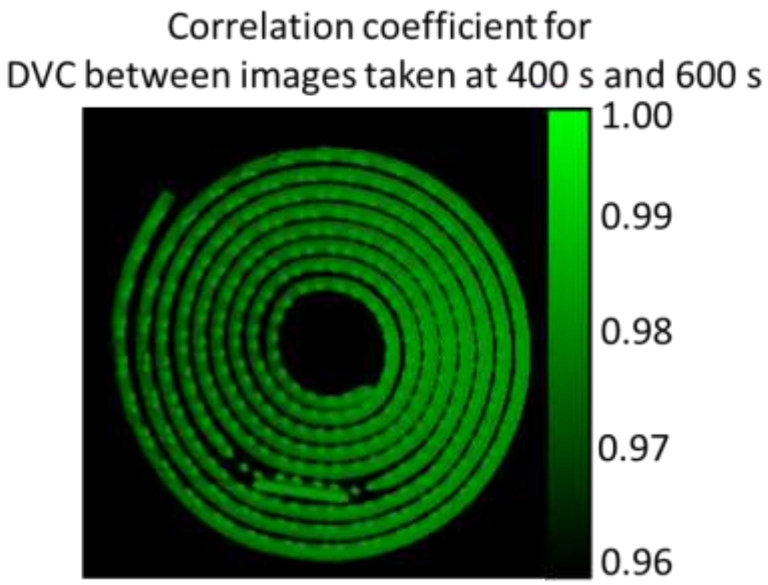

Supplementary Figure 3. *XY* slice from the correlation coefficient profile for DVC between images taken at 400 s and 600 s.

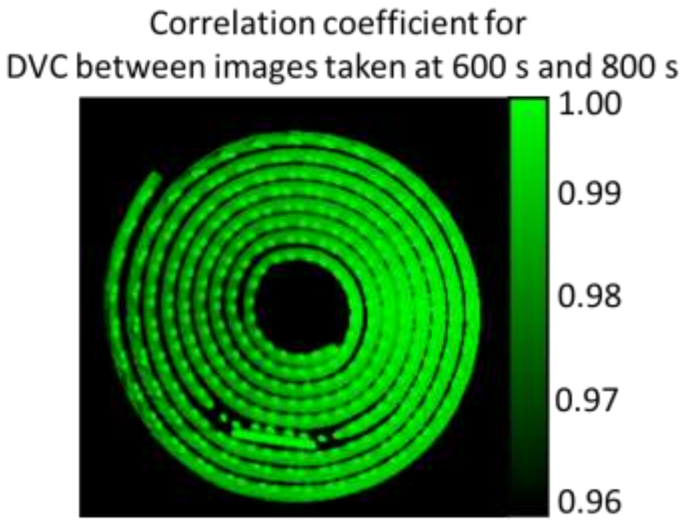

Supplementary Figure 4. *XY* slice from the correlation coefficient profile for DVC between images taken at 600 s and 800 s.

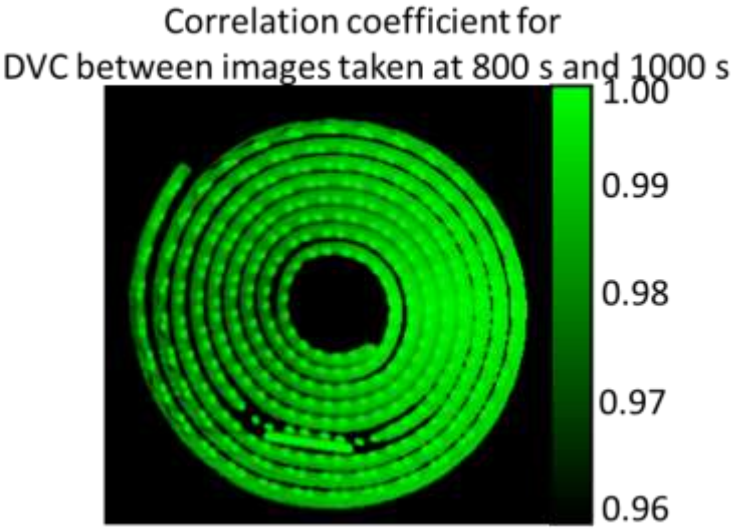

Supplementary Figure 5. *XY* slice from the correlation coefficient profile for DVC between images taken at 800 s and 1000 s.

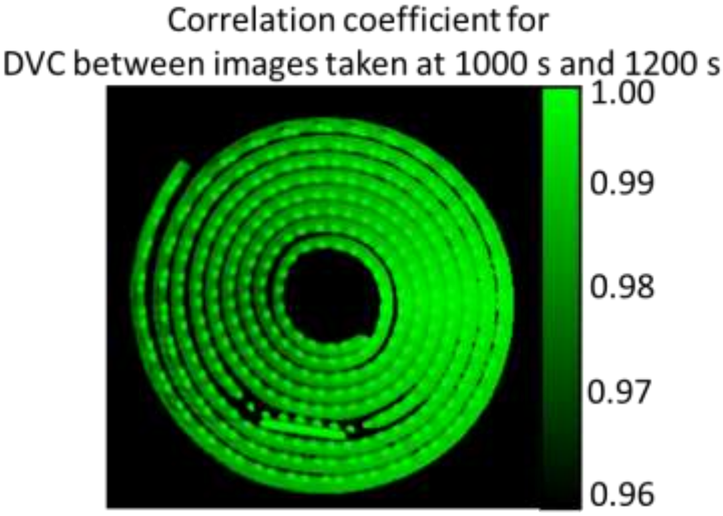

Supplementary Figure 6. *XY* slice from the correlation coefficient profile for DVC between images taken at 1000 s and 1200 s.

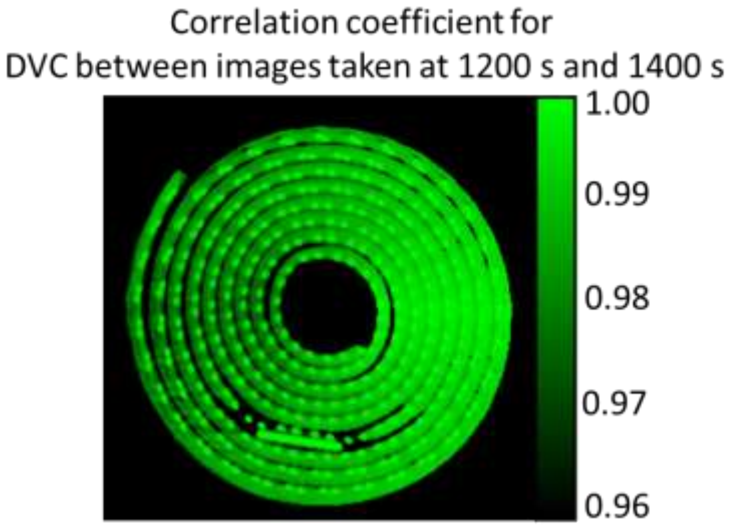

Supplementary Figure 7. *XY* slice from the correlation coefficient profile for DVC between images taken at 1200 s and 1400 s.

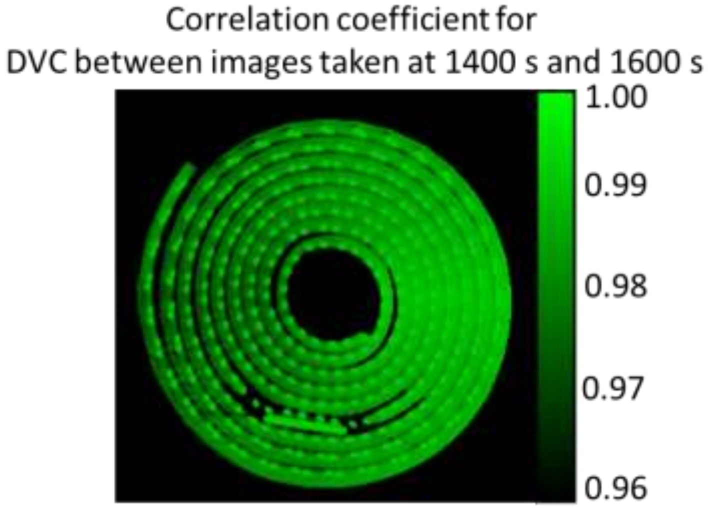

Supplementary Figure 8. *XY* slice from the correlation coefficient profile for DVC between images taken at 1400 s and 1600 s.

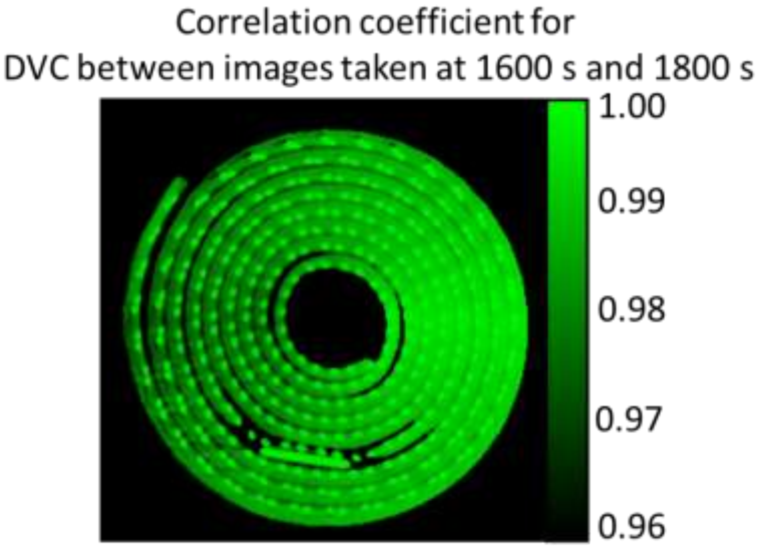

Supplementary Figure 9. *XY* slice from the correlation coefficient profile for DVC between images taken at 1600 s and 1800 s.

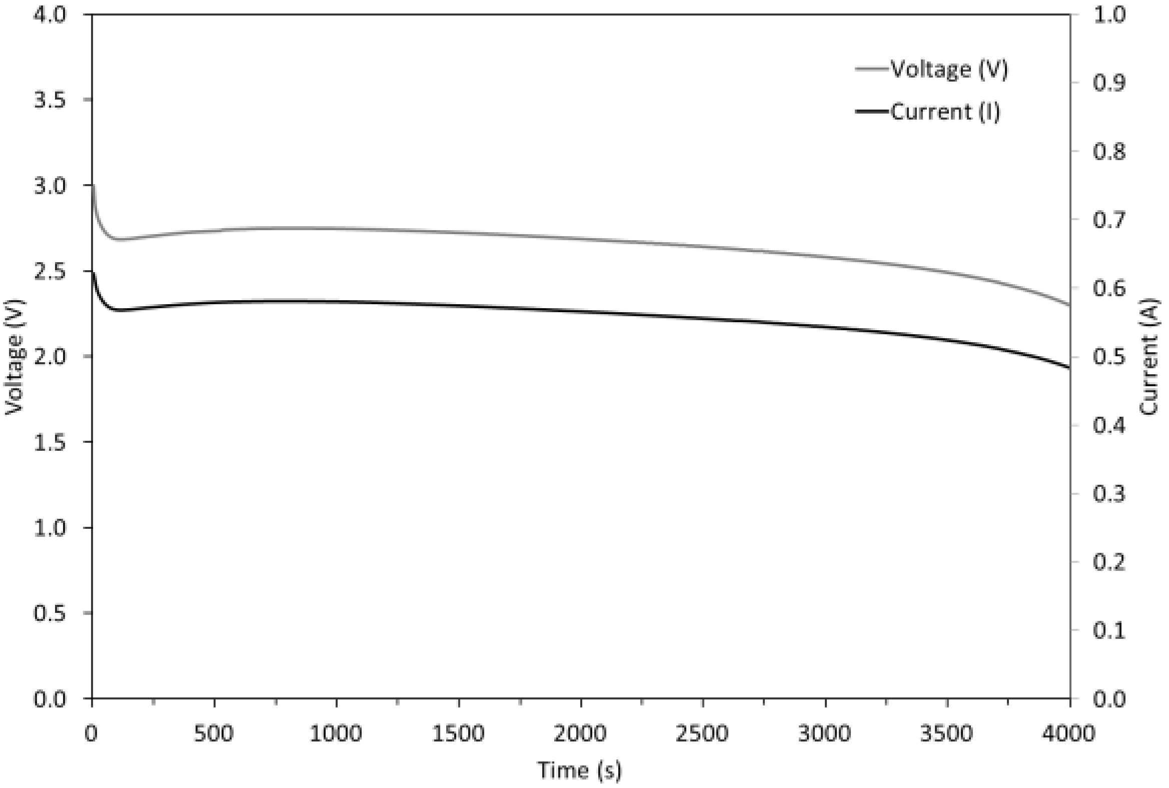

Supplementary Figure 10. Discharge curve of a CR2 cell at constant resistance of 4.5 Ω.
